# Creating a Novel Physical Resilience Measure among Older Adults: The Health, Aging, and Body Composition Study

**DOI:** 10.1093/geroni/igaa057.1578

**Published:** 2020-12-16

**Authors:** Chenkai Wu, Ya-Xi Li, Michelle Odden, Megan Marron

**Affiliations:** 1 Duke Kunshan University, Kunshan, Jiangsu, China; 2 Duke Kunshan University, Kunshan, China; 3 Stanford University, STANFORD, California, United States; 4 University of Pittsburgh, Pittsburgh, Pennsylvania, United States

## Abstract

The concept of resilience has gained increasing attention in aging research; however, current literature lacks consensus on how to measure resilience. We constructed a novel resilience measure based on the degree of mismatch between persons’ frailty level and disease burden and examined its predictive validity. We also sought to explore the physiological correlates of resilience. Participants were 2,457 older adults from the Health, Aging, and Body Composition Study. We constructed the resilience measure as the residual taken from the linear model regressing frailty on age, sex, race/ethnicity, 14 diseases, self-reported health, and number of medications. Participants were classified into three groups—adapters, expected agers, and premature frailers—based on residuals (less than, within, or above one standard deviation of the mean). Validation outcomes included years of able life (YAL), years of healthy life (YHL), years of healthy and able life (YHAL), disability, hospitalization, and survival. The average YHAL was 5.1, 7.7, and 9.1 years among premature frailers, expected agers, and adapters, respectively. Compared with premature frailers and expected agers, adapters had significantly lower rates of disability, hospitalization, and mortality and higher proportion surviving to 90 years. The likelihood of surviving to 90 years was 20.4%, 30.6%, and 39.7% among premature frailers, expected agers, and adapters. We developed and validated a novel approach for quantifying and classifying physical resilience in a cohort of well-functioning white and black older adults. Persons with high physical resilience level had longer healthy life span and lower rates of adverse outcomes.

